# Retrocopied Genes May Enhance Male Fitness

**DOI:** 10.1371/journal.pbio.0030399

**Published:** 2005-10-11

“Retrocopied” genes were long viewed as evolutionary dead ends, with little functional relevance. Such a gene copy is generated by a circuitous route. First, a normal gene is copied to make a messenger RNA, which in the usual scheme of things is sent out of the cell nucleus, used to code for protein, and eventually destroyed. Once in a great while, though, the messenger RNA is “reverse transcribed,” coded back into a DNA sequence by the enzyme reverse transcriptase. It can then be inserted back into a chromosome, quite possibly a different chromosome than the one on which its parent gene resides. This process, which is driven by the legions of transposable elements that litter the genome, usually creates a functionless “retropseudogene,” stranded without a promoter and unable to be expressed.

But occasionally, the new gene copy recruits a promoter by, as yet, largely unknown mechanisms, and may thus potentially become functional, able to code for a protein. In the primate lineage, four such genes have been identified to date. In this issue, Henrik Kaessman and colleagues announce the discovery of seven more, and estimate that on average, one such new gene arises every million years. Many of these genes are expressed predominantly in the testes, where at least some of them probably substitute for X-chromosome genes that are inactivated during sperm development.

The authors first used bioinformatics methods to identify almost 4,000 retrocopies in the human genome. Retrocopied genes can be distinguished from their parents because the introns, or noncoding sequences, of the parent are edited out of the original messenger before retrocopying; thus, the DNA coding sequence is initially the same, but lacks the intervening introns of the parent. Of these 4,000, about 700 had not been disabled by mutations that interrupt the coding sequence. The number of other harmless mutations each had accumulated was then used to estimate the time each retrocopy was formed, based on molecular evolution theory that such neutral mutations occur at a predictable rate. While retrocopies have been created continuously over many millions of years of mammalian evolution, the authors' analysis showed a peak around 40 million years ago, after the emergence of primates, but before establishment of the human line. They estimate that 57 functional retrogenes arose in primates, about one per million years of primate evolution. [Fig pbio-0030399-g001]

**Figure pbio-0030399-g001:**
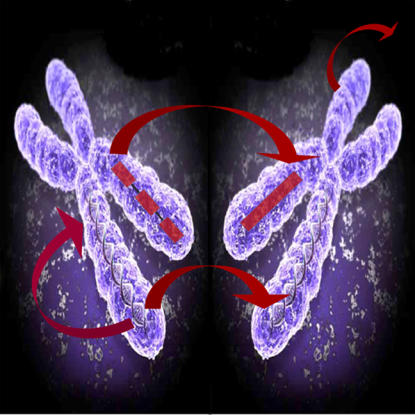
Approximately one new retrogene (a re-inserted copy of a gene) per million years emerged on the primate lineage leading to humans. (Image: © 2005 Hybrid Medical Animation)

To pinpoint individual retrogenes, the authors first conducted an evolutionary simulation to estimate, based on sequence changes, which retrocopies were likely to still be functional. They found seven, which originated between 18 and 63 million years ago. These genes play a variety of roles in transcription and translation, as well as chromosome condensation and segregation, which occur just prior to cell division.

They next looked at expression patterns for these genes in 20 human tissues, and discovered that for all seven, expression was restricted mostly or entirely to the testes. Three of the seven genes were copied from genes on the X chromosome to Chromosome 1, 5, and 12, respectively. The authors suggest that such retrogenes functionally replace their silenced parental genes on the X chromosome during spermatogenesis, a resourceful maneuver that may enhance the reproductive fitness of the organism expressing them. This increase in fitness, in turn, preserves the functional retrocopy through natural selection. For two other genes, the authors also infer a function in spermatogenesis based on the parental gene function. One of these genes as well as the two remaining genes appear to have been selectively driven to evolve new or more adapted functional properties compared to their parents. Together, the results suggest that retrogenes were often recruited during primate evolution to enhance male germline functions. —*Richard Robinson*

